# ‘Fat’s chances’: Loci for phenotypic dispersion in plasma leptin in mouse models of diabetes mellitus

**DOI:** 10.1371/journal.pone.0222654

**Published:** 2019-10-29

**Authors:** Guy M. L. Perry

**Affiliations:** Department of Biology, University of Prince Edward Island, Charlottetown, PEI, Canada; Medical University of Vienna, AUSTRIA

## Abstract

**Background:**

Leptin, a critical mediator of feeding, metabolism and diabetes, is expressed on an incidental basis according to satiety. The genetic regulation of leptin should similarly be episodic.

**Methodology:**

Data from three mouse cohorts hosted by the Jackson Laboratory– 402 (174F, 228M) F_2_ Dilute Brown non-Agouti (DBA/2)×DU6i intercrosses, 142 Non Obese Diabetic (NOD/ShiLtJ×(NOD/ShiLtJ×129S1/SvImJ.*H2*^*g7*^) N_2_ backcross females, and 204 male Nonobese Nondiabetic (NON)×New Zealand Obese (NZO/HlLtJ) reciprocal backcrosses–were used to test for loci associated with absolute residuals in plasma leptin and arcsin-transformed percent fat (‘phenotypic dispersion’; *PD*_*pLep*_ and *PD*_*AFP*_). Individual data from 1,780 mice from 43 inbred strains was also used to estimate genetic variances and covariances for dispersion in each trait.

**Principal findings:**

Several loci for *PD*_*pLep*_ were detected, including possibly syntenic Chr 17 loci, but there was only a single position on Chr 6 for *PD*_*AFP*_. Coding SNP in genes linked to the consensus Chr 17 *PD*_*pLep*_ locus occurred in immunological and cancer genes, genes linked to diabetes and energy regulation, post-transcriptional processors and vomeronasal variants. There was evidence of intersexual differences in the genetic architecture of *PD*_*pLep*_. *PD*_*pLep*_ had moderate heritability ${(h}_{s}^{2}=0.29)$ and *PD*_*AFP*_ low heritability ${(h}_{s}^{2}=0.12)$; dispersion in these traits was highly genetically correlated *r* = 0.8).

**Conclusions:**

Greater genetic variance for dispersion in plasma leptin, a physiological trait, may reflect its more ephemeral nature compared to body fat, an accrued progressive character. Genetic effects on incidental phenotypes such as leptin might be effectively characterized with randomization-detection methodologies in addition to classical approaches, helping identify incipient or borderline cases or providing new therapeutic targets.

## Introduction

Leptin, produced by white adipose cells, regulates appetite, basal metabolic rate, activity, growth and energetic homeostasis, providing negative feedback in the hypothalamus against hunger induced by ghrelin, neuropeptide Y and anandamide [1]. Leptin was first identified as a hunger suppressant in spontaneous hyperphagic *obese* (*ob*) mutant mice (C57BL/KsJ) with glucose intolerance and diabetes [2]. The leptin gene (*Lep*) was mapped to a human leukocyte antigen (HLA) cluster (Chr 6, 29.0 MB) in the *obese* (*ob*) mouse strain [3]. Leptin is linked to T2DM through its role in satiety [1] and to T1DM by glycaemia, insulin sensitivity and triglycerides [4]. Leptin treatment is corrective in animal models of diabetes [5] and leptin polymorphisms have been linked to obesity in humans [6]. Genetic loci have also been linked to leptin production in mice (Chr 3 (143.2 MB), 10 (107.1 MB), 12 (100.6 MB) [7], 7 (~ 100 MB) [8] 14 (37–73 MB) [9], 2 (141.1 MB), 17 (40.2 MB) [10], 5 (93.3 MB), 12 (100.6 MB), 15 (55.3 MB) [11]) and such models may provide further useful information on the genetic structure of diabetes and its physiological construction.

Leptin expression is affected by thyroid or coeliac disease, growth and mass, fatigue and biochemical mediators [5,12,13]. Sex affects leptin physiology in different ways; weight gain is initiated from the deactivation of leptin receptors in the proopiomelanocortin neurons of the arcuate nucleus in females and in males by deactivation of somatotropic leptin receptors [14]. Centrally, however, satiety and hunger are periodic (regulated feeding cycle) or episodic so that the expression of satiety and hunger signals like leptin have both natural entropy [12] and random ultradian pulses [15]. This core feature of hunger suggests that much of the genetic control of leptin production should also be ephemeral or semi-random over time, with some alleles conferring different periodic or episodic expression.

This in turn resembles an emergent phenomenon in which some alleles carry individual variance components in addition to means, distributed as ({*N*_*i*_{*μ*_*i*_,*σ*^2^+*b*}) [16] and termed ‘phenotypic dispersion’ (*PD*) [17] or ‘vQTL’ [18]. Heritable dispersion loci occur in various systems [19] including medical traits. One locus for insulitis dispersion occurs on murine Chr 9 (120.8 MB) [20] linked to *cholecystokinin* (*CCK*) (121.4 MB) [21] and various chemokine receptors. Instability in diabetic phenotype may have epidemiological consequences: the frequency of random hypoglycaemic episodes prior to age five has been associated with reduced long-delay spatial memory [22], for example.

Using three curated mouse mapping datasets (F_2_ Dilute Brown non-Agouti (DBA/2)×DU6i intercrosses [23], female Non Obese Diabetic (NOD)×(NOD×129S1/SvImJ.*H2*^*g7*^) N_2_ backcross [24], and male Nonobese Nondiabetic (NON)×New Zealand Obese (NZO/HlLtJ; T2DM model) backcrosses [11]) hosted by The Jackson Laboratories (ME) I detected several loci for randomized phenotypic dispersion in plasma leptin (*PD*_*pLep*_), several of which were linked to insulitis loci. A possible consensus locus was detected in two cohorts on Chr 17. There was only one locus associated with dispersion in arc-transformed percent body fat (*PD*_*APF*_); morphological traits may be less subject to genetic randomization. Coding polymorphisms at candidate genes linked to the Chr 17 consensus region included those involved with the response to cancer, inflammation, growth, post-transcriptional modification, metabolism and human diabetes incidence. These findings indicate that leptin expression may be partially controlled by randomizing mutations at genes for feeding behaviour and gastrointestinal function, operating on nearly-randomized schedules and undetectable by conventional approaches.

## Materials and methods

### Cohorts

#### Brockmann et al

Data from the original works used in this analysis is hosted by the Mouse Phenome Database, Jackson Laboratories (Bar Harbor, ME; https://phenome.jax.org).

In Brockmann et al., 233 F_2_ DBA/2×DU6i (an inbred subline of DU6 selected for high 6-wk weight (78 generations)) intercross males and 178 females were bred from F_1_ parents from a DU6i sire and a DBA/2 dam (‘Brockmann1’, Cross 2 (https://phenome.jax.org/projects/Brockmann1); MPD:213) [23]. Animals were provided *al libitum* access to a breeding diet (Altromin International #1314; 22.5% protein, 5.0% fat, 4.5% fibre, 6.5% ash, 13.5% water, 48.0% nitrogen-free extract and trace elements and minerals and housed in 350 cm^2^ macrolon type II cages. Fat percentage was calculated from weight and fat mass, and arc-transformed as a proportional value for analysis [25] (‘arcsin-transformed fat percentage’; AFP). Brockmann et al. quantified plasma leptin (ng/ml) using Quantikine Murine ELISA assays (R&D Systems; Weisbaden, DE). Animal welfare in that work was approved by the *Bundesministerium für Ernährung und Landwirtschaft* (BMEL; https://www.bmel.de) (Approval #VI 522 a/7221.3-TV-003/97). F_2_ mice were genotyped at 96 microsatellites (average intermarker spacing = 24.5 MB).

#### Leiter et al

Data from Leiter et al. was based on an NOD/ShiLtJ cross to 129S1/Sv mice (‘Leiter2’, (https://phenome.jax.org/projects/Leiter2); MPD:240). Homozygosity for the diabetogenic NOD MHC *H2*^*g7*^ region is required to produce diabetes in NOD outcrosses [24], so the original investigators bred a homozygous *H2*^*g7*^ 129S1/Sv Chr 17 ‘speed congenic’ line. NOD/ShiLtJ and 129S1/Sv mice were intercrossed; non-MHC markers were fixed by backcrossing to 129S1/Sv males with maximal non-MHC heterozygosity (142 non-MHC microsatellites) and maximal MHC heterozygosity (six generations) [24]. Male 129.*H2*^*g7*^ homozygotes were selected from MHC heterozygotes crosses and bred with NOD/ShiLtJs to create NOD×129.*H2*^*g7*^ F_1_s. These were used to breed 310 female NOD×(NOD×129.*H2*^*g7*^) backcross (BC) mice. Mice were maintained on a 14:10 light:dark photoperiod, irradiated Lab Diet 5LG4 (PMI, Brentwood, MO) and acidified water in order to prevent pathogen exposure [24]. Total weight (‘TW’; g), total lean mass (‘TLM’; g) and total fat (‘TF’; g) were measured by dual-energy X-ray absorptiometry (DXA) and percent fat was calculated from these ((‘PF’ = TF/TW); g) [24]. Genomic DNA was genotyped for 146 SNP, plus at an additional 9–24 SNP around putative diabetes QTL [24] for a total of 308 polymorphic SNP (KBioscience; Hoddeston, UK) (average intermarker distance = 8.3 MB). PLep and AFP were available from 144 mice [24].

#### Reifsnyder et al

In Reifsnyder et al., F_1_ NZO/HlLt×NON/Lt hybrids and NON/Lt mice were used to create 204 NON/Lt×(NZO/HlLt×NON/Lt) backcrosses (‘Reifsnyder1’, (https://phenome.jax.org/projects/Reifsnyder1); MPD:111) [11]. BC mice were held on a 12:12 photoperiod cycle at a controlled temperature and humidity in double plexiglass boxes to the age of 24 weeks, fed NIH-31 grain meal (4% fat) with *ad libitum* access to food and water. Plasma leptin (pLep; ng/ml), body weight (g) and total fat (g) were measured at 24 weeks, with pLep being measured via a commercial radioimmunoassay kit (Linco, Inc.). DNA was isolated from 5mm tail clips or frozen kidney and liver and 83 microsatellite markers (average intermarker distance = 27.0 MB) genotyped using Perkin Elmer or MJ thermocyclers and agarose gels [11]. The Jackson Laboratories Animal Health Program (http://jaxmice.jax.org/genetichealth/health_program.html) ensured the ethical treatment of all animals in the Leiter et al. and Reifsnyder et al. studies. Because of incomplete marker heterozygosity in the DU/6 line after selection, the markers *D3Mit77*, *D5Mit10*, *D8Mit45* and *D18Mit152* were not included in this analysis (see [11]).

### Association analysis

Random genetic effects on pLep and APF were estimated at each locus in each cohort using a type III GLM (Leiter et al.), or mixed model (Brockmann et al., Reifsnyder et al.) [25] in a model of the form

$$y_{ik}=\mu +\alpha_{i}+\beta_{MLH}X_{MLH}+\varepsilon_{ik},$$

where *y*_*ik*_ is trait value, *μ*the cohort mean, *α*_i_ the effect of genotype *i* for each locus *j*, *β*_*MLH*_*X*_*MLH*_ is the partial regression term for the effects of multilocus heterozygosity (MLH) on and *ε*_*ik*_ the OLS residual. Studentized residuals ${\hat{\varepsilon }}_{i\epsilon j}={\hat{\varepsilon }}_{i}/(\hat{\sigma }\sqrt{1-h_{ii}})$ were estimated from OLS residuals in SAS, where $\varepsilon_{ik}=\sum_{i=1}^{m}x_{i}-P$ are divided by the variance of the *i*th residual $var({\hat{\varepsilon }}_{i})=\sigma^{2}(1-h_{ii})$ to control for distributional heterogeneity [26]. MLH was included to account for putative effects of Lerner’s ‘genetic homeostasis’ [27] predicts that random phenotypic aberrancy is a function of internal genetic homogeneity, so that more inbred animals should have a greater rate or degree of phenoabberancy. In order to account for this possible effect, *PD*_*APF*_ and *PD*_*pLep*_ were regressed separately on multilocus heterozygosity (*MLH* = heterozygous loci / total loci) in a non-locus model otherwise as above to test effects of genetic homeostasis as a partial regression covariate. Locus effects were fit to account for the effects of undetected minor QTL. Sex, F_1_ line and/or full sub family were included as appropriate, with full sib family fit as a random effect.

Studentized residuals for each locus were absolute-transformed ${(\hat{|\varepsilon }}_{ik\epsilon j}|)$ to express them as positive vectors of randomization within genotype (‘phenotypic dispersion’; *PD*) [17]. Genotypic variance for *PD* was tested using Tobit quantitative limited models (QLIM) [25] fitting dispersion against *MLH*, full sib family, sex and pedigree where appropriate (as determined from a non-locus model) and for locus effects with a latent variable $y_{i}^{*}$ related to an indicator vector *x*_*i*_ by the quantitative vector *β*, so that $y_{i}^{*}=\beta x_{i}+\upsilon_{i}$ where *υ*_*i*_ *υ*_*i*_ is normal error (*υ*_*i*_ *~ N*(0,σ^2^)). The observed phenotype $y_{i}=y_{i}^{*}$ where latent $y_{i}^{*}>\tau$, and the defined censoring threshold *τ* where $y_{i}^{*}$ does not exceed *τ*. In this system, the lower bound *τ* was set to zero so that $y_{i}=y_{i}^{*}$ where $y_{i}^{*}>0$, and 0 where $y_{i}^{*}\leq 0(L=\sum_{i=1}^{N}f(y)/(1-\phi (\alpha )))$. Joint Wald contrasts were used to determine the significance of marker effects for the Brockmann et al. F_2_ cohort [25] and model *t*-tests for BC cohorts (Leiter et al., Reifsnyder et al.). Additive/dominant genetic architecture for dispersion loci was estimated by general linear contrasts [25] in the Brockman et al. group; this was not in the backcross cohorts since only two genotypes were available. Locus effects were corrected for multiple tests via Benjamini-Hochberg (‘Benj’) correction where *P*-values were ranked 1…*m* so that the largest *P*_*i*_ satisfying the relation *P*_*i*_ ≤ *P*_*k*_ = *k*_*i*_*α/m* (*k* = rank of the *i*th test) was the *α*_*0*.*05*_ experiment-wise error rate [28]. Marker positions in base pairs (BP) were obtained from the GRCm38 Mouse Genome Assembly (https://www.ncbi.nlm.nih.gov/assembly/GCF_000001635.20/).

### Linkage mapping, pLep, APF

Linkage mapping analysis of APF and pLep were performed in the Leiter et al. cohort to compare findings of means effects to those for dispersion; both pLep and APF were already mapped in Brockmann et al. [23] and Reifsnyder et al. [11]. Simple and epistatic effects on pLep in Leiter et al. were mapped in R/QTL [29] using the Cox et al. mouse linkage map [30] at *LOD*_*error*_ > 4.0, 1 cM intervals on a Kosambi function with a maximal error tolerance of *P* < 0.001. Significance thresholds were set using 5000 permutations in R/QTL.

### Linked SNP

SNP at nonsynonymous coding sites, mRNA-untranslated regions (UTR) and splice sites were identified over each range of markers significantly associated with dispersion traits with a minimum ±10 MB window for single markers. Gene identities for SNP were collected from Mouse Genome Informatics (MGI) (www.informatics.jax.org) and and gene functions were interpreted from GeneCards (www.genecards.org), UniProt (www.uniprot.org) and eEnsembl (useast.ensembl.org).

### Heritability

The heritability of *PD*_*APF*_ and *PD*_*pLep*_ was calculated in a set of 43 Mouse Phenome Project strains (n = 1,780) hosted by the Mouse Phenome Database (MPD; http://www.jax.org/phenome) with sample sizes ranging from 4–26 by strain and sex [31] (‘Naggert1’ (https://phenome.jax.org/projects/Naggert1); MPD:143) (Table 1). Studentized residuals and demographic effects on pLep and APF were estimated in the mixed model [25]

$$y_{ij}=\mu +\alpha_{i}+\gamma_{j}+\alpha_{i}\gamma_{j}+\varepsilon_{ijk},$$

where *y*_*ij*_ was the original phenotype, *μ* the experiment-wide leptin mean, *α*_*i*_ the (random) effect of strain, *γ*_*j*_ the (fixed) effect of sex, *α*_*i*_*γ*_*j*_ strain-sex interaction and *ε*_*ijk*_ was error. Studentized residuals were absolute-transformed to *PD*_*pLep*_ and *PD*_*APF*_ as above. Dispersion for each trait ${\hat{|\varepsilon }}_{i\epsilon j}|$ was recoded in PEST4.2.3 [32] for genetic variance/covariance component analysis in VCE5.1.2 [33]. A modified genetic animal model [34] was used to estimate broad strain-level genetic variance $\left(h_{s}^{2}\right)$ and genetic covariance between *PD*_*pLepp*_ and *PD*_*APF*_ in the model **y = Xb + Ya + e**, where **y**_**i**_ is the *n*×*r* matrix for *PD*_*pLep*_ (*r*_1_) and *PD*_*APF*_ (*r*_2_), **X** is a fixed *n*×*p* incidence matrix (0, 1) for *p* nongenetic effects, **b** is the *n*×*p* coefficient vector for unknown fixed nongenetic effects, **Y** is the *i*×*r* incidence matrix for random genetic effects (strain (*i*)), **a** is the *i*×*r* coefficient matrix for random genetic effects (distributed as N~(0, *σ*^2^)) and **e** is the error matrix [35]. Coefficients for strain effects were solved using Gauss-Seidel (GS) iteration and sex by Jacobi iteration. Total strain genetic variance proportions for *PD*_*pLep*_ and *PD*_*APF*_ were calculated as $h_{s}^{2}=\sigma_{s}^{2}/\sigma_{p}^{2}$ (see [36]) and genetic correlation as $r_{a}=\sigma_{g(xy)}/\surd (\sigma_{g(x)}^{2}\sigma_{g(y)}^{2})$.

**Table 1:** Data availability for male (m) and female (f) mice from Svenson *et al*. [31] (‘Naggert1’ (https://phenome.jax.org/projects/Naggert1); MPD:143) with records for serum leptin (‘Lep’, ng/ml) and percent body fat (PF).

Strain	Lep_f_	APF_f_	Lep_m_	APF_m_
129S1/SvImJ	14	8	11	10
A/J	14	10	14	10
AKR/J	9	18	9	17
BALB/cByJ	12	0	10	9
BALB/cJ	14	9	10	10
BTBR	11	10	12	10
BUB/BnJ	9	12	10	8
C3H/HeJ	10	10	10	10
C57BL/10J	14	9	9	12
C57BL/6J	18	10	16	10
C57BLKS/J	17	12	11	10
C57Br/cdJ	16	12	10	10
C57L/J	11	10	10	10
C58/J	18	11	14	9
CAST/EiJ	11	9	10	8
CBA/J	18	10	12	8
CE/J	8	0	18	0
CZECHII/EiJ	12	16	13	11
DBA/1J	10	12	15	7
DBA/2J	10	9	14	9
FVB/NJ	10	9	6	10
I/LnJ	13	9	9	12
JF1/Ms	12	10	17	7
KK/HlJ	10	8	9	9
LP/J	10	9	10	9
MA/MyJ	0	9	10	10
MOLF/EiJ	10	7	9	7
MSM/Ms	12	8	8	12
NOD/ShiLtJ	13	15	10	8
NON/ShiLtJ	13	10	13	10
NZB/BlnJ	8	0	6	10
NZW/LacJ	11	10	11	10
PERA/EiJ	12	9	7	9
PL/J	9	10	10	0
PWK/PhJ	12	7	11	0
RF/J	10	0	8	0
RIIS/J	9	9	9	0
SEA/GnJ	19	9	11	10
SJL/J	10	9	11	15
SM/J	8	9	8	9
SPRET/EiJ	4	4	7	6
SWR/J	14	10	14	10
WSB/EiJ	17	8	13	9

### Protein variants

Coding non-synonymous (CNS) polymorphisms were identified within multiple-cohort consensus genomic locus areas for dispersion in the same trait (*PD*_*pLep*_ or *PD*_*APF*_), fitting the non-diabetic/non-obese line 129SV/SvImJ as a control (‘reference’) strain and the diabetic/obese NOD/ShiLtJ line as the comparison (‘affected’) strain from Leiter et al. [24] using the Genomic Region search function on the Mouse Genomics Informatics (www.informatics.jax.org) [37] platform. DU6–DBA DNA and protein sequence was not available for the DU6 strain [23]; similarly, NON–NZO/HlLtJ polymorphisms were not obtainable since sequence information was not available for the latter [37]. Amino acid sequences for each source strain were extracted based on CNS codon differences for submission to the PredictProtein server meta-service [38], which produces predicted protein structure and activity on a by-amino acid basis for submitted strands. The PROFsec module [39,40] uses a neural network interface to predict squared solvent accessibility scores (predicted accessibility, ‘PACC’) as square Angstroms (Å^2^)) based on minimal atomic bonding distances [41]. Protein-protein, protein-DNA and protein-RNA binding sites were predicted on a by-residue basis using a machine-learning module, ISIS2, using a combination of empirical three-dimensional predictions and curated known activities [42,43]. Finally, binary predictions of polypeptide flexibility were made by residue using the META-Disorder module, which compiles sequence information from into a single two-state (binary 0/1) score along the length of a polypeptide, where flexibility was scored as $B_{norm}=(B-{\bar{B}}_{C\alpha })/\sigma$ > +3 [44,45] (flexible state), where ${\bar{B}}_{C\alpha }$ is the average residue motility based on X-ray chromatography [46]. Only isoforms from well-represented transcribed RefSeq annotations (NM polypeptide accessions/NR RNA accessions) were used to predict polypeptide structure and function; unverified, probable and model sequences (XM/XR annotations) were discounted in constructing haploid protein constructs. For single genes with multiple interstrain CNS identified in MGI, all coding sequence polymorphisms were combined into single strain polypeptide strands for residue analysis.

General gene functions for alleles with CNS polymorphisms between the source strains were obtained from MGI, the Wellcome Sanger Institute (https://www.sanger.ac.uk), the Rat Genome Database (www.rgd.mcw.edu), UniProt (www.uniprot.org), WikiGenes (https://www.wikigenes.org) and NCBI (https://www.ncbi.nih.gov).

## Results

### Genomic homeostasis

*MLH* was significantly correlated with pLep in Leiter et al. (*P* = 0.0007), but not with *PD*_*pLep*_ in any cohort (*P* > 0.3). *MLH* was positively associated with APF in Brockmann et al. (*P* = 0.0083) and marginally in Leiter et al. (*P* = 0.0511). *MLH* was positively associated with *PD*_*APF*_ in Leiter et al. (*β* = 1.00 (SE 0.483), *P* = 0.0379) but not with *PD*_*APF*_ in either other cohort (*P* > 0.5).

### Genetic mapping

APF was transformed as log(arcsin(*APF*))+1 prior to analysis. No standard or epistatic QTL or joint single-locus effects were detected for log(pLep) or log(APF) at the 5% significance threshold in Leiter et al.

### Dispersion analysis

PLep was affected by sex (*F* = 13.7, *P* = 0.0003) and full sib family (*F* = 3.25, *P* = 0.0003). *PD*_*pLep*_ was affected by sex, being significantly lower in females (*β* = -0.335 (SE 0.0687) *t* = -4.89, *P* < 0.0001) and also by family (F = 36.7, *P* = 0.0001). Subfamily effects on *PD*_*pLep*_ were significant in males (*t* = 23.2, *P* = 0.0167) and marginally so in females (*t* = 17.4. *P* = 0.0972). In Reifsnyder et al., pLep was significantly affected by *MLH* (*F* = 11.8, *P* = 0.0007) and pedigree line (*F* = 9.48, *P* = 0.0024).

**Fig 1:**
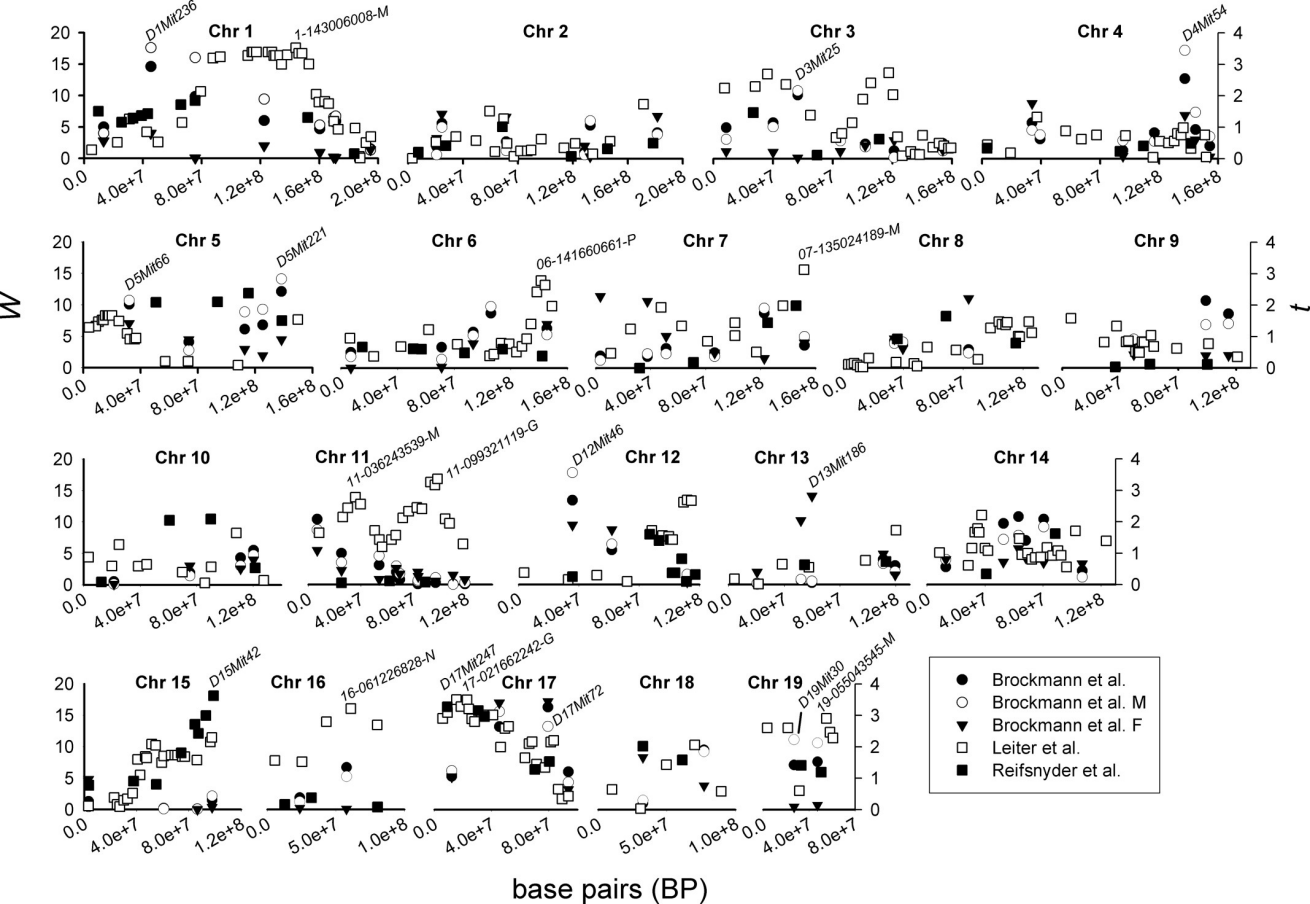
Association mapping of phenotypic dispersion (absolute Studentized residuals from genetic model; ‘*PD*’) in plasma leptin (*PD*_*pLep*_) on murine chromosomes 1–19 Jackson Laboratory in 402 (228M, 174F) F_2_ Dilute Brown non-Agouti (DBA/2)×DU6i intercrosses (Brockman et al.), 144 Non Obese Diabetic (NOD/ShiLtJ×(NOD/ShiLtJ×129S1/SvImJ.*H2*^*g7*^) N_2_ backcross females (Leiter et al.), 204 male Nonobese Nondiabetic (NON)×New Zealand Obese (NZO/HlLtJ) reciprocal backcrosses (Reifsnyder et al.) house mice (*Mus musculus*).

**Fig 2:**
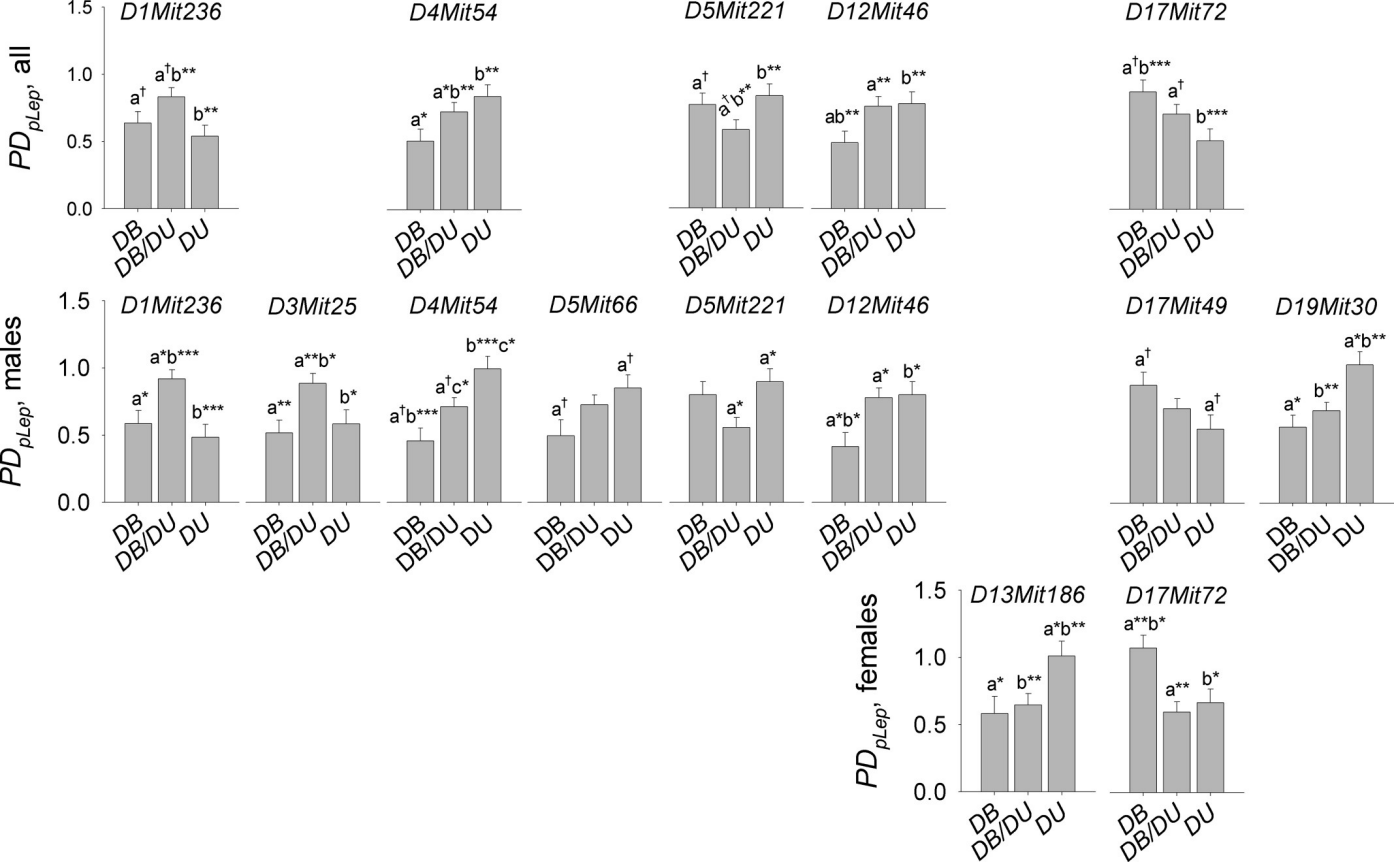
Mean phenotypic dispersion in plasma leptin (*PD*_*pLep*_) from Tobit limited model analysis in a cohort of 233 F_2_ DBA/2× DU6i house mice (*Mus musculus*) (Brockmann et al.) by marker. The significance of differences among mean *PD*_*pLep*_ by genotype (*P* < 0.05*, < 0.01**, < 0.001***) was determined using general linear contrast statements.

*D1Mit236*, *D4Mit54*, *D5Mit221*, *D12Mit46* and *D17Mit72* were associated with *PD*_*pLep*_ (*P*_*Benj*_ < 0.05) in the Brockman et al. cohort (Fig 1; Table 2). *D4Mit54* and *D17Mit72* were additive in construction (*P*_*cont*_ < 0.001) (Fig 2). *D1Mit236* appeared overdominant (significantly higher *PD*_*pLep*_ in DBA/2×DU6i heterozygotes) (*P*_*cont*_ < 0.001) and *D5Mit221* underdominant (significantly lower *PD*_*pLep*_ in DBA/2×DU6i heterozygotes) (*P*_*cont*_ < 0.01) in the complete population. *PD*_*pLep*_ in DBA/2×DU6i heterozygotes and DU6i homozygotes at *D12Mit46* (Fig 2).

**Table 2:** Genomic marker peaks for dispersion in plasma leptin (*PD*_*pLep*_) and arcsin-transformed percent body fat (*PD*_*APF*_), plasma in 402 (174F, 228M) F_2_ Dilute Brown non-Agouti (DBA/2)×DU6i intercrosses (Brockman et al.), 142 female Non Obese Diabetic (T1DM model; NOD/ShiLtJ)×(NOD/ShiLtJ×129S1/SvImJ.*H2*^*g7*^) N_2_ backcross female mice (Leiter et al.), and 204 male Nonobese Nondiabetic (NON)×New Zealand Obese (NZO/HlLtJ; T2DM model) reciprocal backcrosses (Reifsnyder et al.) at Benjamini-Hochberg-adjusted significance thresholds. The Brockman cohort was divided into male (M) and female (F) mice. Cross type, number, chromosome, genetic marker, marker chromosomal location (base pairs; bp), nominal *P*-value, proportion of total variance in *PD*_*pLep*_, genetic architecture (‘Arch’; A = additive, D = (positive) dominant, D- = negative dominant, OD = overdominant, UD = underdominant [37]), contrast test coefficients (*β* (SE)) and the significance of architecture interpretations as estimated from contrast tests (*P*_*arch*_). Contrast coefficients for Leiter et al. indicates average increase in *PD*_*pLep*_ in NOD/ShiLtJ×129S1/Sv heterozygotes over NOD/ShiLtJ homozygotes and for Reifsnyder et al. *PD*_*pLep*_ in NON homozygotes compared to NZO/HlLtJ heterozygotes.

Cohort	Type	N	Chr	Marker	BP	*P*	r^2^	Arch	*β* (SE)	*P*_*arch*_
Brockmann	F_2_	353	1	*D1Mit236*	45,435,532	0.0007	0.032	OD	0.485 (0.136)	0.0004
			4	*D4Mit54*	137,446,526	0.0017	0.030	A	0.331 (0.0982)	0.0008
			5	*D5Mit221*	138,397,460	0.0023	0.026	UD	-0.439 (0.139)	0.0018
			12	*D12Mit46*	35,725,744	0.0013	0.030	D	0.561 (0.160)	0.0005
			17	*D17Mit72*	79,393,017	< 0.0001	0.035	A	-0.363 (0.0982)	0.0002
Brockmann–M	F_2_	202	1	*D1Mit236*	45,435,532	0.0002	0.073	OD	0.763 (0.185)	< 0.0001
		202	3	*D3Mit25*	56,600,668	0.0046	0.056	OD	0.671 (0.191)	0.0006
		201	4	*D4Mit54*	137,446,526	0.0002	0.084	A	0.537 (0.132)	< 0.0001
			5	*D5Mit66*	32,048,625	0.0047	0.029	A	0.357 (0.148)	0.0169
			5	*D5Mit221*	138,397,460	0.0009	0.042	UD	0.586 (0.195)	0.0030
			12	*D12Mit46*	35,725,744	0.0001	0.048	D	0.751 (0.237)	0.0018
		197	17	*D17Mit72*	79,393,017	0.0004	0.031	A	0.326 (0.137)	0.0185
			19	*D19Mit30*	26,884,618	0.0038	0.044	D-	0.807 (0.229)	0.0005
Brockmann–F	F_2_	159	13	*D13Mit186*	59,775,079	0.0009	0.071	D-	-0.794 (0.236)	0.0010
		159	17	*D17Mit72*	79,393,017	0.0002	0.092	D-	-0.881 (0.226)	0.0002
Leiter	BC	142	1	1-143006008-M	143,991,782	0.0004	0.080	-	0.378 (0.108)	-
		139	6	06-141660661-P	141,480,730	0.0055	0.053	-	0.309 (0.111)	-
		142	7	07-135024189-M	151,765,715	0.0017	0.065	-	0.338 (0.109)	-
		142	11	11-036243539-M	35,942,588	0.0054	0.052	-	-0.303 (0.109)	-
		140	11	11-099321119-G	98,342,842	0.0008	0.075	-	-0.355 (0.106)	-
		139	16	16-061226828-N	60,950,000	0.0013	0.069	-	0.310 (0.111)	-
		138	17	17-021662242-G	22,989,572	0.0005	0.081	-	-0.370 (0.106)	-
		138	19	19-055043545-M	55,103,853	0.0037	0.056	-	-0.315 (0.109)	-
Reifsnyder	BC	201	15	*D15Mit42*	98,887,109	0.0003	0.051	-	0.305 (0.0842)	-
		200	17	*D17Mit247*	9,168,430	0.0011	0.053	-	-0.261 (0.0797)	-

*PD*_*pLep*_ loci and their architecture were highly similar in F_2_ DBA/2×DU6i males to those detected in the complete cohort, with *D3Mit25* (overdominant), *D5Mit66* (additive) and *D19Mit30* (negative dominant; DU6i homozygotes having higher *PD*_*pLep*_ than other genotypic classes) also being associated with *PD*_*pLep*_ (*P*_*Benj*_ < 0.05) (Figs 1 and 2; Table 2). *D17Mit72* was significantly associated with *PD*_*pLep*_ in female F_2_ DBA/2×DU6i intercrosses but this locus was negative dominant in females (*P* < 0.001). *D13Mit186* also had a negative dominant association with *PD*_*pLep*_ in female F_2_s (Figs 1 and 2; Table 2). No significant marker-by-sex interaction was found using two-way mixed interactive models (*P*_*Benj*_ > 0.1) so that differences in structure by sex were likely scalar rather than interactive [47]. Variance in plasma leptin was over twice as high in males ($\sigma_{m}^{2}$ = 25.0) as females ($\sigma_{f}^{2}$ = 9.8) in Brockmann et al., but variance in fat percentage by sex was roughly equivalent ($\sigma_{m}^{2}$ = 0.448, $\sigma_{f}^{2}$ = 0.460). Of all *PD*_*pLep*_ loci in males and females, three were over- or underdominant (*D1Mit236*, *D3Mit25* and *D5Mit221*), one was dominant (*D12Mit46*), two were additive (*D4Mit54* and *D5Mit66*), two negative dominant (*D13Mit186* and *D19Mit30*) and one which was additive in males but negative dominant in females (*D17Mit49*) (Fig 2; Table 2).

Positions on Chr 1, 6, 7, 11, 16 and 17 were associated with *PD*_*pLep*_ in the Leiter et al. cohort (Fig 1; Table 2) while *D15Mit42* and *D17Mit247* were associated with *PD*_*pLep*_ in Reifsnyder et al. (*P*_*Benj*_ < 0.05). Genetic architecture could not be calculated in these cohorts since backcrosses differentiate only the homozygote and heterozygote state. Pedigree group was not associated with *PD*_*pLep*_ (*P* > 0.05). Chr 17 markers for *PD*_*pLep*_ in Leiter et al. and Reifsnyder et al. were linked (23.0 and 9.2 MB, respectively) but neither was strongly linked to the Brockmann et al. Chr 17 locus (45.5 MB and 79.4 MB in males and overall, respectively). Chr 19 loci from Brockmann et al. and Leiter et al. were more distal (26.9 MB and 55.1 MB, respectively) (Fig 1; Table 2).

Only a single SNP marker, 06-141660661-P, from the Leiter et al. cohort was associated with *PD*_*APF*_ at the *P*_*0*.*05*,*BH*_ threshold with lower dispersion in NOD/Lt×129S1/SvImJ.*H2*^*g7*^ heterozygotes (*β* = -0.329 (SE 0.0882), *P* = 0.0002, *r*^2^ = 0.0884).

### Genetic variance/covariance

Males had marginally higher *PD*_*pLep*_ than females (*μ*_m_ = 0.629 SE 0.0705; *μ*_f_ = 0745; SE 0.0705; *P* < 0.0807) in the Naggert et al. collection. Strain (*P* < 0.0001; $\sigma_{G}^{2}/\sigma_{p}^{2}$ = 0.217), and sex-by-strain interaction (*P* < 0.0001; $\sigma_{Gxs}^{2}/\sigma_{p}^{2}$ = 0.094) both were significant modifiers of *PD*_*pLep*_ (Table 2). Sex did not affect *PD*_*APF*_ (*P* > 0.7), and main effects of strain were only marginally significant (*P* = 0.0837; $\sigma_{G}^{2}/\sigma_{p}^{2}$ = 0.058), although sex-by-strain interaction effects were significant (*P* = 0.0002; $\sigma_{Gxs}^{2}/\sigma_{p}^{2}$ = 0.099).

**Fig 3:**
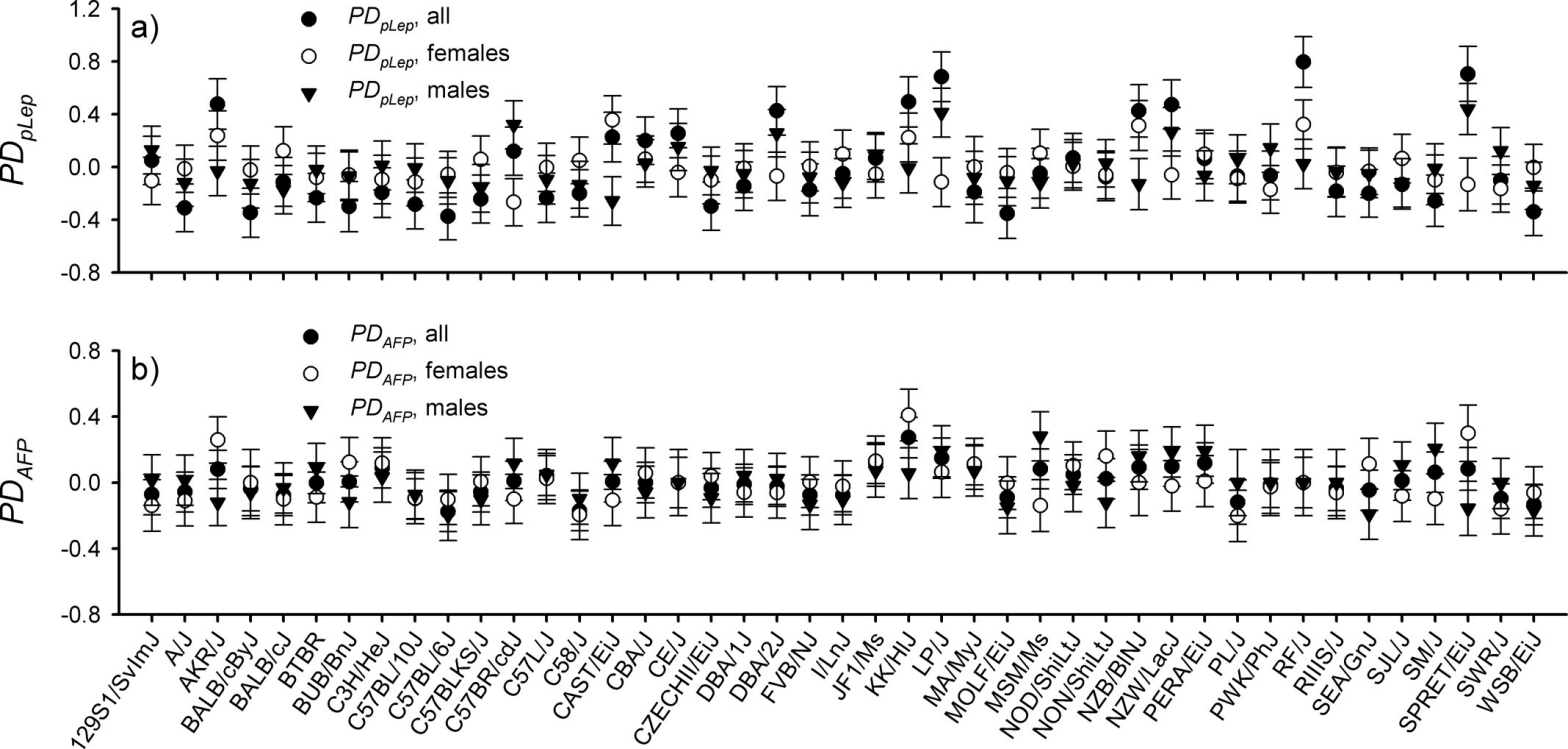
Strain-by-sex means for phenotypic dispersion in a) plasma leptin (*PD*_*pLep*_) and arcin-transformed percent body fat (*PD*_*APF*_) in all (solid circles), female (empty circles) and all (solid downwarn-facing triangles) from 43 mouse strains (n = 1,780; n_i_ = 4–26 per strain and sex) [31] using mixed models [25].

Of all observations, 298 had observations for both traits, 669 for *PD*_*pLep*_ only and 457 *PD*_*APF*_ only. Heritability ${(h}_{g}^{2})$ for *PD*_*pLep*_ was 0.293 (SE 0.0323) $\sigma_{g}^{2}$ 0.172 (SE 0.0348)) and for *PD*_*APF*_ was 0.122 (SE 0.0244) ($\sigma_{g}^{2}$ 0.0502 (SE 0.0176)), and the strain-level genetic correlation (*r*_*g*_) was 0.796 (SE 0.0723) (*σ*_*pLep*,*APF*_ 0.0741 (SE 0.0240)) (*L* = 1480). *PD*_*pLep*_ means were highest in the AKR/J, DBA/2J, KK/HlJ, LP/J, NZB/BlNJ, NZW/LacJ, RF/J and SPRET/EiJ lines (Fig 3).

### Protein variants

A single dispersion locus consensus area was called for *PD*_*pLep*_ on Chr 17 (1–22,989,572 BP) from the Leiter et al. cohorts. NM RefSeq CNS variants within this area were identified using MGI and indexed by accession number and location (S1 Table). CNS polymorphisms were detected at i) *T cell lymphoma invasion and metastasis 2* (*Tiam2*), which distorts allele transmission and expression [48] and regulates neurite growth [49]; ii) *NADPH oxidase 3* (*Nox3*), which forms reactive oxygen species (ROS) by catalyzing electron transfer from NADPH to O_2_ [50] and has been linked to T2DM in West Africans [51]; iii) *zinc finger*, *DHHC domain containing 14* (*Zdhhc14*), which is overexpressed in gastric tumours [52] and lymphoproliferation [53]; *synaptojanin 2* (*Synj2*), an important lipid phosphatase involved in vesicle recycling [54,55]; iv) *fibronectin type III domain containing 1* (*Fndc1*) involved with squamous and basal cell carcinomas [56]; v) *brachury 2* (*T2*), a *t-complex* gene critical to the development of the embryonic axis [57]; vi) *phosphodiesterase 10A* (*Pde10A*) which mediates intracellular signal transduction by hydrolyzing intracellular cAMP and cGMP concentrations [58], linked to bipolarity [59], the suppression of which is linked to diabetes and obesity [60] via thermoregulation [61]; vii) *poly (A) binding protein*, *cytoplasmic 6* (*Pabpc6*), which polyadenylates the tail of mRNA precursors [62]; viii) *insulin-like growth factor 2 receptor* (*Igf2r*); ix) *adherens junction formation factor* (*Afdn*), an Ca^2+^-independent target of *Ras* that helps create cell-cell adhesions [63]; x) *hyaluronan synthase 1* (*Has1*), which synthesizes hyaluronan (tissue homeostasis, provides compression resistance and lubricates tissues) in connective tissues and recruits lymphocytes in cancerous tissues [64]; and xi) an array of vomeronasal and zinc-finger genes (S1 Table).

Overall, there was little to no differentiation in predicted accessibility (PACC) or binary inferences of disorder across transcript types, except for some minor shifts in PACC between *Synj2* b haplotypes near polymorphism sites (S1 Fig). However, most polypeptide sequences had from 1–5 differences in the presence or position of protein-protein binding sites (*i*.*e*. *Synj2* c, e, *Fndc1*, *Pde10a*, *Papbc6*, *Vmn2r96*, *Vmn2r111*), usually within 50–100 residues of the CNS polymorphism (S1 Fig).

## Discussion

Association analysis indicated several loci for leptin dispersion in these three cohorts, with a possible consensus locus for *PD*_*pLep*_ in the anterior area of Chr 17 (9.1–23.0 MB) in N_2_ NOD/ShiLtJ×(NOD/ShiLtJ×129S1/SvImJ.*H2*^*g7*^ backcross mice (Leiter et al.) and in unrelated NON/Lt×(NZO/HlLt×NON/Lt) backcrosses (Reifsnyder et al.) on Chr 17, suggesting possible synteny, with an additional locus occurring in both sexes on Chr 17 (79.3 MB) in Brockman et al. There also appeared to be sex differences in the genetic structure of leptin dispersion loci on Chr 1 (45.4 MB), 4 (137.5 MB), 5 (138.4 MB), 12 (35.7 MB) and Chr 19 (26.9 MB). Other works have found mostly negative dominant architectures for dispersive loci in medical traits (diabetes, albuminuria) [65,66], but in this work genetic architecture was divided overall between additivity, dominance and heterosis. The backcross structure in the Leiter et al. and Reifsnyder et al. cohorts may have limited the detection of dominant effects. In addition to genetic differences among the source strains themselves, the marked differences in rearing environment, age and methodology among the three cohorts here could limit the ability to detect consensus loci or alter apparent genetic construction either in trait means or random dispersion effects. Summed variance proportions for loci in Brockmann et al. and Leiter et al. approximated gross estimates of heritable variance in dispersion ($h_{g}^{2}$ = 30%) from the Naggert et al. strain collections. *PD*_*pLep*_ loci were unlinked to normal murine leptin QTL (Chr 2: 141.4 MB [10]; Chr 3: 14.2 MB; Chr 10: 106.5 MB; Chr 14: 37.2 MB [7]; Chr 7: 100 MB [8]) with the exception of the 17B3 (~ 44.3 MB) *PD*_*pLep*_ locus [10]) but five of eight *PD*_*pLep*_ peaks from the Leiter et al. dataset were linked to insulitis QTL from that same study (Chr 1: 121 MB; Chr 11: 42 and 114 MB; Chr 17: 24 MB; Chr 19: 50 MB) [24].

Genotypes causing instability or lability in leptin production would fit with the profile of the inherent variability (periodic or episodic pulses) in plasma leptin [15,67]) according to satiety, so that heritable dispersion could well be an integral, mathematical-physiological facet of serum leptin. Significant random or apparently variation occurs in several other diabetic traits (*i*.*e*. glycaemia [68], CAPN10 [69], suggesting that periodic or episodic physiological variation might be a common feature of diabetes in general as a normal feature of hunger and gut emptying. Either form of dysregulation–excessive variability or physiological inflexibility–could be a precursor to irregular reactions to hunger, resulting in inappropriate or incorrectly controlled feeding behaviour. Such loci might thus reflect the various positions of individuals on the longer-term onset to full diabetes, or affect repeatability among analyses (*i*.*e*. [7,70]. This may be less true for accruing morphological characters like obesity, where adipose mass probably reflects complex, long-term leptin-diet relationships, leptin resistance and feedback [71,72].

Analytical solutions incorporating dispersive effects might help physiological uncertainties in diabetes. A database search (NCBI, RGD) found no identified QTL for amylin production but the Chr 6 *PD*_*pLep*_ peak in Leiter et al. (141.5 MB) co-located with coding and UTR SNP at *amylin* (*islet amyloid polypeptide* (*Iapp*)) (142.3 MB), a leptin agonist and insulin/glucagon regulator which forms pancreatic amyloid processes with cytotoxic effects on pancreatic β cells in T2DM [73]. Agonistic amylin-leptin expression might operate reactively via randomization with incidental satiety or hunger touching off cycles of randomized counter-regulation.

The general basis of dispersive gene action–whether through genes with core physiological functions or those directly related to a given dispersed trait–is unclear [66]. Coding polymorphisms in the consensus Chr 17 area included those linked to cancer (*Fndc1*, *Tiam2*, *Zdhhc14 Has1*) [48,52,56,64], growth and development (*Igf2r*, *Afdn*, *Tiam2*, *T2*) [49,57,63], immunology [53] or diabetes itself via the energetic electron transfer chain (*Tiam2*, *Pde10A*) [50,51] or basal thermal metabolism [61]. *Tiam2* modifies allele transmission and expression [48] in addition to its other roles, which could be a central modifier of the propensity to dispersive or stable physiological function. *Pabpc6* was another possible core candidate linked to the consensus Chr 17 locus via its role in poly-A post-translational processing [62]. Alternatively, dispersion in leptin could be related to coding variants at the various vomeronasal Chr 17 genes; neurological pathway alleles affecting lability in food detection could similarly be involved with randomization in leptin production upstream or downstream of sensory components of feeding activity. Notably, SNP at *Fndc1* were linked to loci associated with dispersion in urine albumin [66].

### Fat

Only a single locus was detected for *PD*_*APF*_ and heritability for dispersion in fat from Naggert et al. was low ($h_{s}^{2}$ = 0.12). Morphological characters with physical benchmarks achieved on stable, progressive trajectories like weight or total fat proportion might be less susceptible to dispersion. An assay of 38 mouse mapping cohorts ($\bar{x}$ = 133.2 markers, *n* = 238 mice/cohort, *n*_*T*_ = 13,571) found relatively few loci for dispersion in body weight (Perry, unpub), suggesting that overall morphology is relatively immune to heritable randomization. As an anabolic process, morphological indicators of diabetes may be a result of numerous ontogenetic corrections to achieve an integrated final value for body proportion [74] commensurate with overall genetic and environmental proclivity to obesity.

### Sex

Male/female differences in the expression of dispersion loci occurred in F_2_ DBA/2×DU6i mice: only the Chr 17 45.4–79.4 MB locus was common to males and females, and both *PD*_*pLep*_ loci in females were negative dominant. Architecture at that locus also varied between males (additive) and females (negative dominant). Sex-based differences in the quantitative genetic structure of disease traits is common [75], including insulin resistance in mouse models [76], leptin resistance [77] and the biochemical operation of leptin [14,78]. Heritable sex-related differences in leptin pulses might be as integral to diabetes onset as conventional means and biochemical action. Women experience greater signal amplitude in leptin expression [67], which could be partially determined by greater severity of dispersive gene action. Correspondingly, the percent variance (*r*^2^) of residuals associated with locus effects was slightly higher in female F_2_s from Brockmann et al. (8.2%) than for males (5.1%). Leptin dispersion might even be a component of greater leptin resistance in males [77], with uncontrolled variance in leptin being ignored by a static, unresponsive physiognomy.

### Genetic homeostasis

*MLH* was marginally positively correlated with *PD*_*APF*_ in the Leiter et al. set, but was unassociated with other dispersed traits in these groups. In accordance with the predictions of genetic homeostasis [27], *MLH* is usually negatively associated with dispersion [66] (Perry, unpub) so that more inbred individuals tend to be more phenotypically divergent from the mean. The marginal association of MLH with *PD*_*APF*_ may simply be likely random chance.

## Conclusion

Loci for randomized variance in plasma leptin is in line with the notion of periodic or episodic variation in satiety, and the linkage of several leptin dispersion loci to diabetes susceptibility loci (see [24]) suggests a role for physiological randomization in diabetes physiology with insulitis itself. Dispersive effects on core diabetic traits like leptin production might help explain heterogenous presentation, progression and response to treatment (see [8,79]); of the nearly 400 million sufferers of diabetes mellitus, many incipient affecteds are unaware of their condition [80]. Quantification of heritable randomization in the structure of underlying diabetic phenotype might help elucidate both genetic labilities and liabilities, helping resolve unassigned statistical variance in the underlying elements of diabetic physiology.

## Supporting information

S1 Table List of 129S1/SvImJ (129)-*vs*-NOD/ShiLtJ (NOD) single nucleotide polymorphisms (SNP (dbSNP Build 142)) from Leiter et al. [24] linked to *PD*_*pLep*_ by mouse chromosome (Chr), co-ordinate (base pair (BP) position), MGI gene ID, standard symbol, mutation type (coding non-synonymous (CNS), splice site (SS), noncoding transcript variant (NTV), mRNA-UTR (UTR)) and genotype in each strain. (XLS)

S1 Fig Predicted accessibility (PACC; Å^2^)) (solid wavy lines; Y-axis), binary disorder (solid horizontal lines) and binding sites (solid symbols = NOD/ShiLtJ, open symbols = 129S1/SvImJ.*H2*^*g7*^; protein-protein binding sites = triangles, protein-DNA sites = squares, protein-RNA sites = circles) for candidate genes in the Chr 17 consensus *PD*_*pLep*_ region by polypeptide from Leiter et al. [24] and Reifsnyder et al. [11] estimated on the PredictProtein meta-platform [38] based on recognized and curated inter-strain coding polymorphisms obtained from Mouse Genomics Informatics (www.informatics.jax.org) [37]. Where single lines are present for PROFbval scores, predicted polypeptide-sequence accessibility was the same for both genotypes. Interstrain differences in coding sequence are indicated by SNP sequence id at the top of each subfigure using open downward-facing triangles.
(JPG)
