# Supplementary figures and images for: ‘Fat’s chances’: Loci for phenotypic dispersion in plasma leptin in mouse models of diabetes mellitus
Source: PLoS One. 2019 Oct 29;14(10):e0222654. doi: 10.1371/journal.pone.0222654 (PMC6818960; doi:10.1371/journal.pone.0222654)

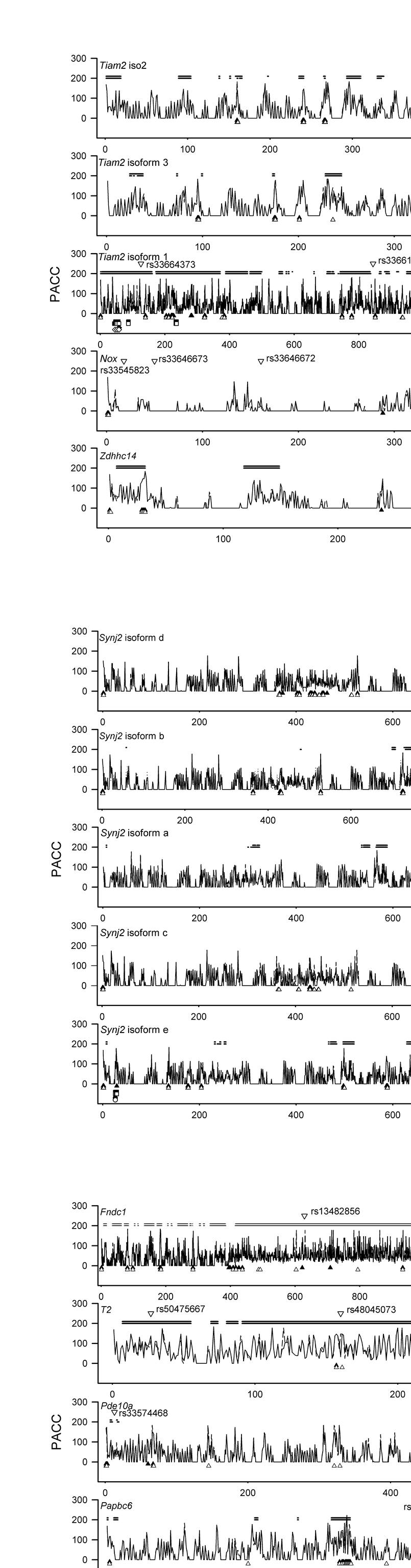

Supplement: S1 Fig — Where single lines are present for PROFbval scores, predicted polypeptide-sequence accessibility was the same for both genotypes. Interstrain differences in coding sequence are indicated by SNP sequence id at the top of each subfigure using open downward-facing triangles. (JPG) [file pone.0222654.s002.jpg]
